# Staged multimodal correction of implant rippling after prepectoral breast reconstruction with thin mastectomy flaps: a case report

**DOI:** 10.1080/23320885.2026.2739821

**Published:** 2026-09-30

**Authors:** Tony Kazzi, Suzanne Youness, Antoine Abi Abboud, Hady Rihani

**Affiliations:** Division of Plastic and Reconstructive Surgery, University of Balamand, Beirut, Lebanon

**Keywords:** implant rippling, prepectoral breast reconstruction, subpectoral conversion, fat grafting, cohesive gel implant

## Abstract

Rippling is a recognized aesthetic complication of prepectoral implant-based breast reconstruction, and thin mastectomy flaps markedly increase its risk. We report a 35-year-old physically active woman with recurrent bilateral fibroadenomas who underwent bilateral nipple-sparing subcutaneous mastectomy with immediate prepectoral implant reconstruction; the prepectoral plane was chosen to avoid animation deformity, weighed against her thin flaps. Visible rippling attributable to thin soft-tissue coverage developed at three months. Autologous fat grafting gave only partial, temporary correction. Four months later, a single staged, combined procedure achieved definitive correction: bilateral exchange to highly cohesive silicone gel implants, conversion to a subpectoral pocket, Wise-pattern mastopexy, and adjunctive lipofilling, with a right local inferior-pedicle rotation flap correcting a medial contour defect. At 12 months, rippling had resolved with a satisfactory aesthetic outcome. Thin flaps were considered the principal contributing factor, although a single case cannot establish causation. Because prepectoral reconstruction relies on the soft-tissue envelope for implant camouflage, thin flaps may favor considering primary subpectoral or dual-plane placement, or planned soft-tissue support such as acellular dermal matrix or autologous coverage. Salvage addresses the determinants of rippling in combination: fat grafting to thicken tissue, pocket conversion to add muscular coverage, mastopexy to tighten the skin envelope, and exchange for a more cohesive implant, although simultaneous performance precludes isolating any single component’s contribution. Thin mastectomy flaps appear to be an important risk factor, potentially informing patient selection. Once rippling occurs, a staged multimodal approach can restore contour.

## Introduction

Fibroadenomas are among the most common benign breast tumors and, when numerous, recurrent, or associated with deformity, may occasionally warrant subcutaneous nipple-sparing mastectomy to achieve definitive treatment and avoid repeated interventions [1–3]. In such cases the reconstructive challenge is essentially aesthetic, and the quality of the soft-tissue envelope becomes the principal determinant of outcome.

Implant-based reconstruction may be performed in a prepectoral or subpectoral plane, each with distinct advantages and complications [4,5]. Prepectoral placement preserves the pectoralis major and can produce a natural contour, but because it lacks muscular coverage it depends heavily on the thickness and quality of the overlying soft tissue [5,6]. Implant rippling—visible or palpable wrinkling of the implant shell through the skin—is therefore a well-recognized complication of prepectoral reconstruction, and its incidence rises substantially in patients with thin mastectomy flaps [6,7].

Reported rates of rippling after implant-based augmentation or reconstruction vary widely, from 0% to 35% across published series, reflecting differences in soft-tissue thickness, implant characteristics, and grading criteria [7]. Once established, rippling is difficult to correct and frequently requires more than one strategy, including fat grafting, implant exchange for a more cohesive device, pocket conversion, mastopexy, or the addition of soft-tissue support such as acellular dermal matrix or autologous flaps [7,8]. However, the literature seldom documents the complete trajectory of a single patient from the development of rippling, through an initial incomplete correction, to definitive multimodal salvage.

The aim of this report is twofold. Clinically, we describe a staged, multimodal salvage of persistent bilateral rippling after prepectoral reconstruction in a patient with thin mastectomy flaps. Conceptually, we use this trajectory to emphasize a preventive lesson that is often understated in the rippling literature: appropriate patient selection—specifically, recognizing thin mastectomy flaps as a relative contraindication to unsupported prepectoral placement—is the most effective way to avoid this complication in the first place.

## Case presentation

A previously healthy 35-year-old woman with a history of two pregnancies presented with bilateral multiple fibroadenomas of the breasts (Figure 1). Her body-mass index was 25 kg/m^2^, the breast skin was of good quality without significant striae, and she had mild breast ptosis (Regnault grade I–II). She had undergone several prior excisional procedures at the ages of 15, 21, and 27 at another institution to remove symptomatic lesions. Despite these interventions, multiple fibroadenomas persisted in both breasts.

**Figure 1.**
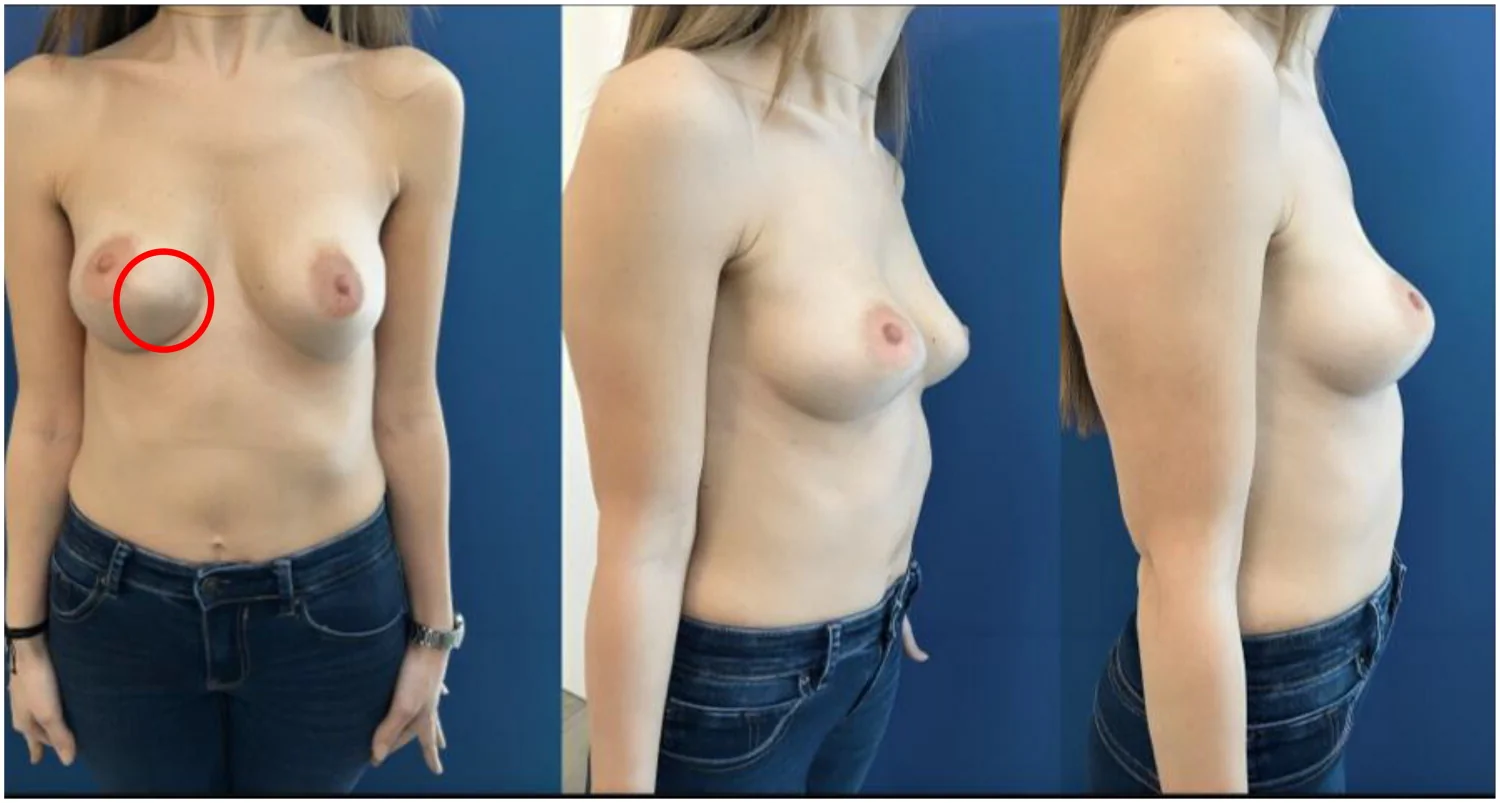
Preoperative clinical photographs demonstrating bilateral breasts with multiple fibroadenomas. Anterior, oblique, and lateral views are shown, with a prominent fibroadenoma highlighted by a red circle.

After comprehensive evaluation and in collaboration with the obstetrics and gynecology team, a decision was made to perform bilateral nipple-sparing subcutaneous mastectomy to remove the remaining fibroadenomas while preserving the nipple–areola complex. A structured summary of the clinical course is provided in Table 1.

**Table 1.** Clinical timeline.

Time point	Clinical event	Intervention
Ages 15, 21, 27	Recurrent bilateral fibroadenomas	Three prior excisional procedures (outside institution)
Age 35 (day 0)	Persistent multiple bilateral fibroadenomas	Bilateral nipple-sparing subcutaneous mastectomy; immediate prepectoral reconstruction (Mentor High Profile 400 cc) + minimal lipofilling
2 weeks	Satisfactory contour and symmetry	Clinical observation
3 months	Visible implant rippling; cosmetic dissatisfaction	Autologous fat grafting (abdominal harvest, local anesthesia)
∼7 months (4 months after grafting)	Persistent rippling (particularly right breast) and mild ptosis	Definitive staged revision planned
∼7 months	Definitive revision	Bilateral: implant exchange to highly cohesive gel (Mentor MemoryGel Xtra 405 cc), prepectoral→subpectoral conversion, Wise-pattern mastopexy, popcorn capsulorrhaphy, adjunctive lipofilling; right breast inferior-pedicle rotation flap for medial defect
12 months post-revision	Complete resolution of rippling; satisfactory result	Follow-up

### Initial operative procedure

The subcutaneous mastectomy was performed by the obstetrics and gynecology team through inframammary incisions, preserving a thin layer of subdermal tissue to maintain vascular supply to the skin envelope and nipple–areola complex. The reconstructive portion was then performed by the plastic surgery team. Immediate bilateral reconstruction was achieved with silicone implants (Mentor High Profile, 400 cc; microtextured surface) placed in the prepectoral plane, with minimal lipofilling to correct minor contour irregularities. No acellular dermal matrix or synthetic mesh was used, and no surgical drains were placed. The mastectomy flaps were subjectively assessed as thin at operation; flap thickness was not quantitatively measured. The prepectoral plane was elected through shared decision-making: the patient was physically active and specifically wished to avoid the animation deformity associated with subpectoral placement, and this preference was weighed against the recognized thinness of her flaps, with autologous fat grafting planned as a means of augmenting soft-tissue coverage should contour irregularity develop. Two weeks postoperatively the patient demonstrated satisfactory contour and symmetry (Figure 2). The early postoperative course was uneventful, without infection, seroma, hematoma, or wound-healing complications.

**Figure 2.**
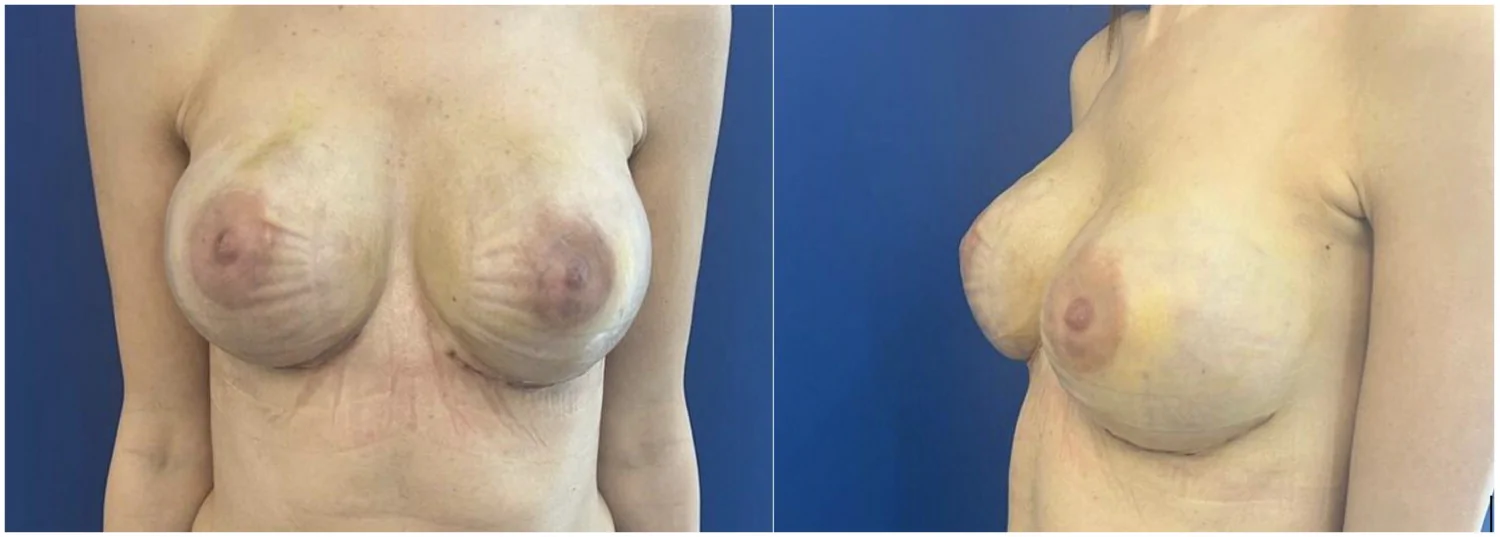
Clinical photographs two weeks after bilateral nipple-sparing mastectomy with immediate prepectoral implant reconstruction, showing good contour and symmetry. Superficial skin markings are due to bra compression.

### Postoperative course and lipofilling

Three months after reconstruction, the patient developed visible implant rippling with cosmetic dissatisfaction (Figure 3). To increase soft-tissue thickness and improve implant camouflage, autologous fat grafting was performed under local anesthesia. Fat was harvested from the abdomen using 10-mL syringes, processed, and reinjected through 2-mm cannulas [9]. Overcorrection was performed to account for an estimated 30% graft resorption, and injection was discontinued once the recipient tissue reached saturation to reduce the risk of fat necrosis. The total volume of fat injected per breast was not recorded. Although lipofilling improved the soft-tissue envelope and reduced the visibility of the implant edges, the effect was partial and temporary; persistent rippling remained four months later, particularly in the right breast (Figure 4).

**Figure 3.**
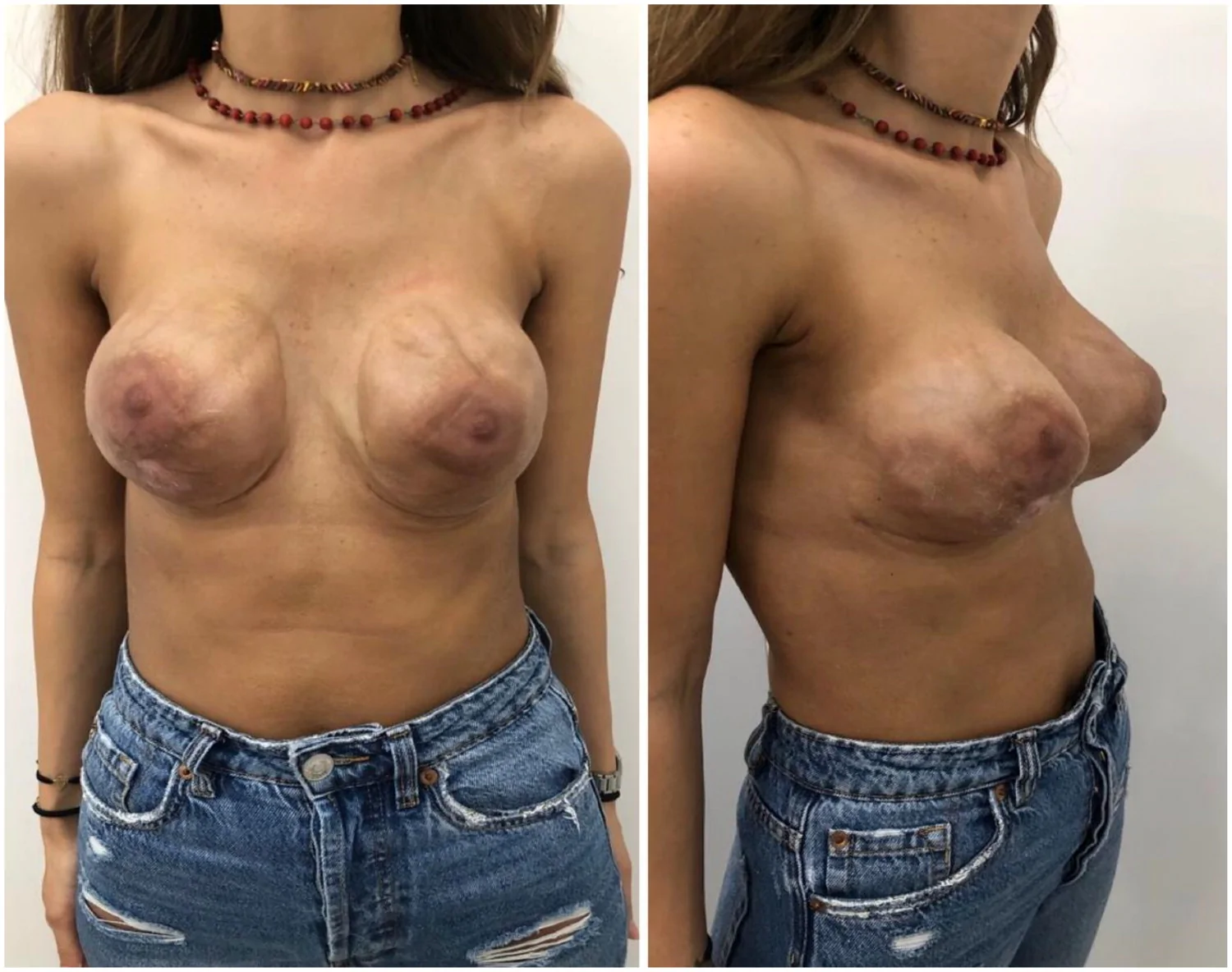
Clinical photographs three months after prepectoral reconstruction demonstrating visible implant rippling.

**Figure 4.**
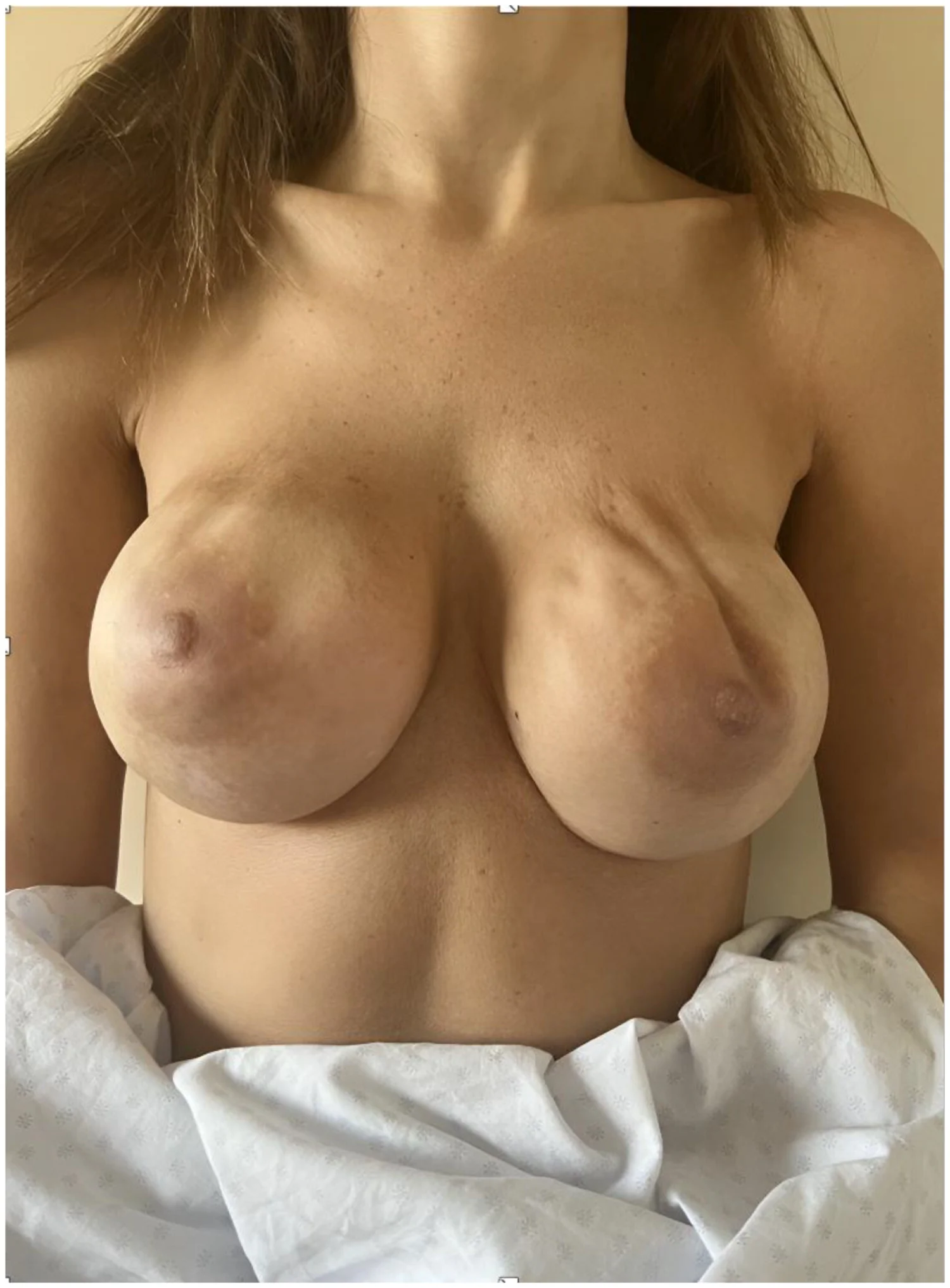
Clinical photograph four months after autologous fat grafting. Despite improved soft-tissue coverage, persistent implant rippling is visible, particularly in the right breast.

### Revision surgery

Because of persistent rippling and mild ptosis, definitive revision was undertaken with implant exchange, pocket conversion, and mastopexy.

#### Right breast

An incision was made along the previous scar approximately 1 cm above the inframammary fold. Dissection was carried to the prepectoral pocket and the existing implant was removed. Popcorn capsulorrhaphy was performed to tighten the oversized prepectoral capsule. The prepectoral capsule was appreciably larger and more voluminous on the right than on the left; this asymmetry mirrored the more pronounced rippling and the medial contour defect on the right and was consistent with a slightly larger right-sided pocket and greater soft-tissue laxity on that side. Capsulorrhaphy was therefore required on the right but not on the left. A new pocket was created in the subpectoral plane beneath the pectoralis major, and a highly cohesive silicone gel implant (Mentor MemoryGel Xtra, high-profile, 405 cc) was inserted. A Wise-pattern mastopexy with a superior pedicle was performed to remove excess skin and improve contour. To correct a depression along the medial aspect of the right breast, an inferior-pedicle flap was de-epithelialized, mobilized, rotated medially, and secured with 3-0 Vicryl sutures; adjunctive lipofilling was performed concurrently to further soften the implant edges.

#### Left breast

The same approach was applied to the left breast—implant exchange, conversion to the subpectoral plane, Wise-pattern mastopexy, and adjunctive lipofilling—without the need for an inferior-pedicle rotation flap on this side.

The combined bilateral revision was completed in approximately 3 h of operative time. No drains were placed, and the patient recovered uneventfully, without infection, hematoma, seroma, skin or nipple–areola necrosis, or wound-healing problems. At 12-month follow-up the patient demonstrated a satisfactory aesthetic result with improved contour and complete resolution of the previously visible rippling (Figure 5).

**Figure 5.**
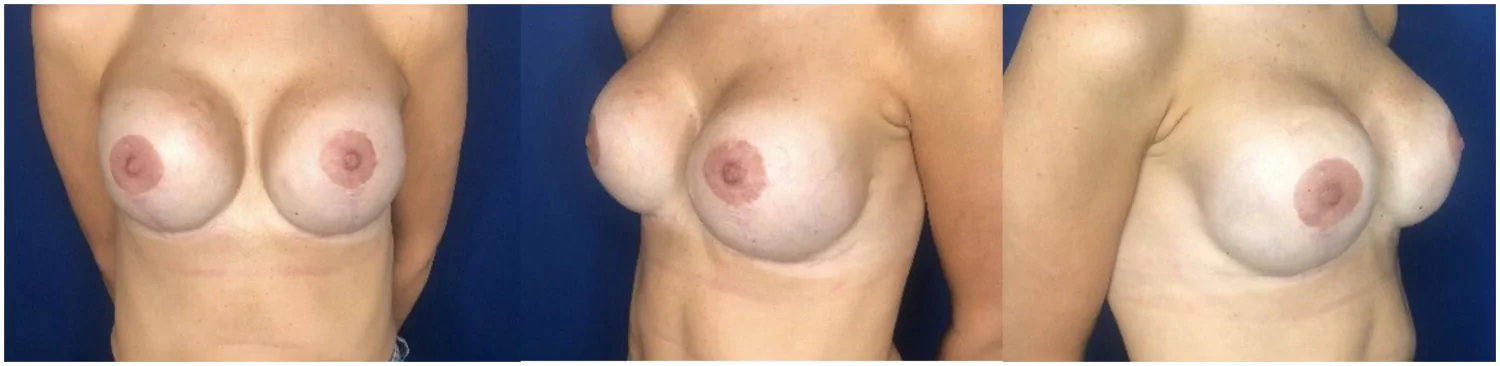
Postoperative clinical photographs at 12-month follow-up (anterior and lateral views) demonstrating improved contour and complete resolution of the previously visible rippling.

### Patient perspective

The patient reported that the visible rippling had been a source of considerable distress and self-consciousness, compounded by her long history of repeated breast operations. She noted that the initial fat grafting produced some improvement but did not resolve her concerns, and that she remained dissatisfied until the definitive revision. At final follow-up she reported being very satisfied with the contour and symmetry of her breasts and felt the outcome met her expectations.

## Discussion

Rippling is a well-recognized complication of implant-based reconstruction and reflects visible wrinkling of the implant shell through insufficient overlying soft tissue [7]. Its principal determinants are implant characteristics, pocket size and plane, surgical technique, and—most importantly in prepectoral reconstruction—the thickness of the soft-tissue envelope [5–8]. In our patient, thin mastectomy flaps following removal of the glandular tissue were considered the most likely principal contributing factor; because flap thickness was not measured, this remains a clinical impression rather than an objectively quantified finding.

### Patient selection: the first line of defense

The most important lesson of this case is preventive. Prepectoral reconstruction depends on the soft-tissue envelope for implant camouflage, so patients with thin or devascularized mastectomy flaps are precisely those in whom unsupported prepectoral placement is least forgiving [5,6]. In such patients, primary subpectoral or dual-plane placement provides an additional muscular layer over the implant and may reduce the likelihood of contour deformity from the outset [5]. Where a prepectoral plane is preferred, robust soft-tissue support should be planned in advance—either an acellular dermal matrix, as described by Tettamanzi et al. in a fixation technique associated with reduced complications [10], or autologous coverage such as the dermo-adipose flap over a polyurethane implant reported by Lembo et al. in skin-reducing mastectomy [11]. Careful implant selection and precise pocket dimensioning are equally important to avoid a skin–implant size mismatch that predisposes to rippling. Had the thinness of the flaps been given greater weight at the index operation, a primary subpectoral or soft-tissue–supported approach might have avoided the subsequent revisions altogether. Because these observations derive from a single patient, they should be regarded as hypothesis-generating rather than as a practice recommendation.

### Salvage strategy once rippling is established

When rippling does occur, correction is often staged. Autologous fat grafting is a minimally invasive first-line option that increases tissue thickness and smooths visible implant edges [9], and it produced partial, temporary improvement in our patient. When it proved insufficient, conversion to a subpectoral pocket added a muscular layer between the implant and skin, which may reduce implant visibility and has additionally been associated with lower rates of capsular contracture [5,12]. Wise-pattern mastopexy addressed mild ptosis and tightened the skin envelope, improving implant support [13], and a local inferior-pedicle rotation flap corrected a focal medial depression on the right. Finally, exchange to a highly cohesive silicone gel implant contributed structural stability: more cross-linked gels better maintain shape and resist the shell folding that becomes visible through thin tissue, and higher cohesivity has been associated with less rippling and fewer revisions in prepectoral reconstruction [14,15].

### Attribution and novelty

Because the definitive procedure combined several maneuvers simultaneously—implant exchange, pocket conversion, mastopexy, lipofilling, and a unilateral local flap—this single case cannot establish which component was most responsible for the final result, and causality should not be assigned to any one technique. The value of the report is not a claim that any single maneuver is decisive, nor that multimodal correction is itself novel. Rather, it lies in documenting the complete, sequential trajectory of one patient—from the development of rippling on thin flaps, through an incomplete fat-grafting correction, to definitive salvage—and in drawing from that trajectory the preventive, selection-based lesson that is often underemphasized when rippling is discussed primarily as a problem to be corrected.

### Limitations

This report describes a single patient treated by one surgical team, which limits generalizability. Outcomes were assessed subjectively through clinical examination and photography; no objective contour metrics or validated patient-reported outcome measures (for example, the BREAST-Q) were used, and the patient perspective, while recorded, was not formally quantified. As noted above, the simultaneous nature of the definitive procedure precludes any inference about the relative contribution of individual components. Longer follow-up would be needed to confirm the durability of the correction. In particular, mastectomy flap thickness was not measured intraoperatively, so the central role attributed to flap thinness rests on qualitative clinical assessment rather than objective measurement, which tempers the strength of the patient-selection inference; baseline covariates such as BMI, skin quality, and ptosis grade are reported but were not analyzed as part of a controlled comparison.

## Conclusion

Implant rippling after prepectoral reconstruction is driven chiefly by inadequate soft-tissue coverage, and thin mastectomy flaps appear to be an important risk factor that may influence patient selection. Recognizing this at the outset—favoring subpectoral or dual-plane placement, or planning robust soft-tissue support when a prepectoral plane is used—is the most effective way to prevent the complication. When rippling nonetheless occurs, a staged, individualized, multimodal approach combining fat grafting, subpectoral conversion, mastopexy, and a highly cohesive implant can restore contour and achieve a durable, satisfactory result, although the contribution of any single technique cannot be isolated in a single case.

## Data Availability

The data supporting the findings of this case report are not publicly available due to privacy and ethical restrictions concerning patient-identifiable clinical information. De-identified data are available from the corresponding author (T. Kazzi) upon reasonable request.
